# Lactate levels in severe malarial anaemia are associated with haemozoin-containing neutrophils and low levels of IL-12

**DOI:** 10.1186/1475-2875-5-101

**Published:** 2006-11-06

**Authors:** Climent Casals-Pascual, Oscar Kai, Brett Lowe, Mike English, Thomas N Williams, Kathryn Maitland, Charles RCJ Newton, Norbert Peshu, David J Roberts

**Affiliations:** 1 Nuffield Department of Clinical Laboratory Sciences and National Blood Service Oxford Centre, Oxford, OX3 9DU, UK; 2 Nuffield Department of Paediatrics, Oxford, OX3 9DU, UK; 3 Centre for Tropical Medicine, Nuffield Department of Clinical Medicine John Radcliffe Hospital, Oxford, OX3 9DU, UK; 4 KEMRI Centre for Geographic Medicine Coast, PO Box 230, Kilifi, Kenya; 5 Department of Paediatrics, Faculty of Medicine, Imperial College of Science Technology and Medicine, Exhibition Road, London SW7 2AZ, UK; 6 Neurosciences Unit, Institute for Child Health, 30 Guilford Street, London WC1N 1EH, UK; 7 National Blood Service, Oxford Centre, John Radcliffe Hospital, Headley Way Oxford, OX3 9BQ, UK

## Abstract

**Background:**

Hyperlactataemia is often associated with a poor outcome in severe malaria in African children. To unravel the complex pathophysiology of this condition the relationship between plasma lactate levels, parasite density, pro- and anti-inflammatory cytokines, and haemozoin-containing leucocytes was studied in children with severe falciparum malarial anaemia.

**Methods:**

Twenty-six children with a primary diagnosis of severe malarial anaemia with any asexual *Plasmodium falciparum *parasite density and Hb < 5 g/dL were studied and the association of plasma lactate levels and haemozoin-containing leucocytes, parasite density, pro- and anti-inflammatory cytokines was measured. The same associations were measured in non-severe malaria controls (N = 60).

**Results:**

Parasite density was associated with lactate levels on admission (*r *= 0.56, *P *< 0.005). Moreover, haemozoin-containing neutrophils and IL-12 were strongly associated with plasma lactate levels, independently of parasite density (*r *= 0.60, *P *= 0.003 and *r *= -0.46, *P *= 0.02, respectively). These associations were not found in controls with uncomplicated malarial anaemia.

**Conclusion:**

These data suggest that blood stage parasites, haemozoin and low levels of IL-12 may be associated with the development of hyperlactataemia in severe malarial anaemia.

## Background

*Plasmodium falciparum *malaria is an important cause of global morbidity and mortality. In severe malaria metabolic acidosis is one of the most important determinants of survival [1]. In African children with malaria, the clinical syndrome of respiratory distress usually reflects an underlying metabolic acidosis associated with lactic acidaemia [2]. This syndrome is an important independent, clinical prognostic marker for poor outcome [3].

The pathophysiology of metabolic acidosis is complex. The direct contribution of *P. falciparum *to the final lactate concentration, through anaerobic glycolysis in the parasite itself, is likely to be small [4]. More significantly, an inadequate supply of oxygen to tissues may follow from severe anaemia and provoke a metabolic shift within host cells to anaerobic glucose metabolism and increased lactic acid production. In addition, the flow of blood through the microcirculation may be impeded by adherence of infected erythrocytes to the endothelium of post-capillary venules and/or increased rigidity of uninfected cells [5]. Lactate may not in itself be sufficient to cause acidaemia but the inhibition of oxidative metabolism in the context of an ongoing inflammatory response will cause protons (H^+^) to accumulate and eventually lead to metabolic acidosis [2]. These pathophysiological pathways suggest that the syndrome of lactic acidosis may be associated with the total parasite burden during acute infection.

Classically, parasitaemia has been associated with the severity of clinical disease [6]. However, the relationship is weak and the association of parasite density with specific syndromes of severe disease is less clear. Haemozoin (Hz) or malaria pigment, the final product of digested host haemoglobin, is often seen in circulating leucocytes and may be a surrogate marker for acute or chronic parasite load [7].

However, the clinical significance of Hz has only been investigated quite recently. Nguyen and colleagues found an association between Hz-containing neutrophils (HCN) and outcome and between HCN and Hz-containing monocytes (HCM) and hyperparasitaemia, shock and hypoglycaemia [8]. In African children with severe malaria, Hz containing leucocytes were associated with severe malaria [9,10], cerebral malaria [11] and anaemia [10,12]. More recently, Casals-Pascual and colleagues have reported the association of HCM, free Hz and bone marrow Hz with severe malarial anaemia [13]. However, the relationship of Hz containing leukocytes and lactic levels in malaria has not been described.

Severe disease has also been associated with high levels of pro-inflammatory cytokines. Raised levels of TNF-α and IFN-γ are more frequently observed in children suffering from severe malarial disease than those suffering from mild disease or those with asymptomatic infections (reviewed in [14]). On the other hand, high levels of IL-10 have been associated with protection from anaemia [15]. Finally, low levels of IL-12 have been found in children with severe, compared to mild disease [12,16] and IL-12 may promote a Th1 type response and have other regulatory functions in modulation of an inflammatory response. The immune response to parasites may contribute not only to parasite clearance and amelioration of disease but also to immunopathology and physiological disturbance.

In addition, the relationship between the parasites, cytokines and outcome of infection may depend on the direct effect(s) of Hz on leucocytes. Hz-containing macrophages from the peripheral circulation have increased secretion of inflammatory cytokines [17] or anti-inflammatory cytokines [18]. Moreover, the number of Hz-containing monocytes is associated with serum TNF-α levels in children with malaria [12].

This study has tested the hypothesis that parasitized erythrocytes and/or their products, including Hz and the cytokine response in children with malaria may contribute to lactic acidosis in a series of children admitted with complicated or severe malaria admitted to the ward or to the paediatric intensive care unit suffering from severe malarial anaemia.

## Materials and methods

The study was carried out in Kilifi District Hospital, Kilifi, Kenya. The epidemiology of malaria in Kilifi District has been described elsewhere [3]. Parents of children with a primary diagnosis of malaria with fever any asexual *P. falciparum *parasitaemia and anaemia were invited to participate in the study, which was part of an ongoing study of the relationship of cytokines and malarial pigment to malarial anaemia and erythropoiesis. Prior treatment with antimalarials before admission was an exclusion criterion. Consent was obtained in the local language (Kiswahili or Kigiriyama). The National Ethical Committee, Kenya gave ethical approval for the study.

A three-ml venous blood sample was collected into EDTA and processed immediately. A full blood count was obtained by a haematology analyzer (Coulter^® ^MD II, Coulter Corporation, Miami, Florida). Plasma lactate (both L- and D- isomers) was measured by lactate oxidase activity (Analox Instruments). Peripheral blood films were stained with May-Grünwald-Giemsa.

The number of Hz-containing monocytes per 500 monocytes (HCM) and Hz-containing neutrophils per 500 neutrophils (HCN) were recorded from 3% Giemsa stained thick films. Light microscopy (without polarised light) was used to count HCNs and HCMs. Monocytes and neutrophils were counted as HCM or HCN where these cells contained at least 2 dots of malaria pigment. Microscopists were blinded to any other results. The absolute numbers of HCMs and HCNs were calculated using the number of circulating monocytes and neutrophils, respectively. Plasma concentrations of TNF-α, IL-10, IFN-γ, IL-12 were measured by ELISA (R&D Systems, Abingdon, United Kingdom).

Children with malaria and Hb < 5 g/dl who were symptomatic (namely exhibiting deep breathing, intercostal muscle recession, prostration, or lethargy), had hyperparasitaemia (>50/500 RBCs) or had oxygen saturation < 90%, were transfused (20 ml/kg of whole blood). All children were given parenteral anti-malarials and supportive care according to local clinical protocols. Weight-for-age Z-scores (WAZ) were calculated with EPINUT Anthropometry software (EPIINFO version 6). Blantyre coma score [19] was used to assess the degree of impaired consciousness.

### Statistical analyses

The values of lactate, parasitaemia, TNF-α, IL-10, IL-12, IFN-γ, HCMs and HCNs were normally distributed when log-transformed. A Pearson's correlation was used to measure the linear association between the variables studied. Partial correlations were calculated to control for parasitaemia, number of HCNs and Hb concentration.

Hyperlactataemia was defined as lactate concentration >5 mmol/L. Parasite and host-related variables were compared in children with and without hyperlactataemia using the non-parametric Mann-Whitney test and Kruskal-Wallis for comparisons with more than one group.

The association of HCMs, HCNs and cytokines was investigated with dependent variables (i.e. lactate levels on admission) using linear regression. The variables that failed to show a linear association with lactate levels were not included in the regression analysis.

The scatter plots of variables that showed a linear association were checked for outliers (dFBeta >2/√N), points of high leverage (Cook's distance) and normality of residuals (SPSS 11.0, SPSS Inc., Chicago, Illinois).

## Results

### Population description

26 children with severe anaemia and acute malaria were studied. The main clinical features are described in Table 1. None of these children died during the follow up period (up to 3 months). At 1 month follow-up the mean Hb concentration for these children was 10.3 (SD 1.6) g/dL.

**Table 1:** Population description

		Lactate < 5 mM (N = 14)	Lactate > 5 mM (N = 12)
Age (yrs)	Median (IQR)	2.68 (1.25–4.87)	1.46 (0.9–3.0)
Male/female	ratio	0.75	0.71
Transfused	(%)	35.7	50
Impaired consciousness (BCS ≤ 2)	(%)	0	25
Respiratory distress	(%)	0	25
Parasite density (/μL)*	Median (IQR)	1,625 (170–25,527)	163,200 (61,800–242,655)
WAZ (Z-score)	Median (IQR)	-2.01 (-3.6 to -4.87)	-2.54 (-3.5 to -1.31)
Lactate (mM)	Median (IQR)	2.3 (1.8–3.2)	6.8 (6.4–9.15)
Haemoglobin (g/dL)	Median (IQR)	4.05 (3.7–4.6)	3.8 (3.5–4.3)
MCV (fL)	Mean (95%CI)	68.3 (58.1–76.8)	73.6 (65.8–79.3)
HCN (No/500 PMN)*	Median (IQR)	6 (3–9)	16 (10–19)
HCM (No/500 MNC)	Median (IQR)	25 (11–84)	69 (28.5–84.8)
TNF-α (pg/mL)	Median (IQR)	12.4 (7.8–21.0)	27.5 (15.2–40.6)
IL-10 (pg/mL)*	Median (IQR)	39.2 (22.4–52.3)	116.7 (68.3–231.5)
IFN-γ (pg/mL)	Median (IQR)	2.19 (1.5–3.1)	3.0 (2.4–4.2)
IL-12 (pg/mL)*	Median (IQR)	233.15 (198.1–278.3)	130.4 (87.5–184.8)

The clinical characteristics of 26 children studied. The interquartile range (IQR) or the 95% confidence intervals are given. * *P *< 0.05 (Mann-Whitney test)

### Plasma lactate and parasitological and immunological variables

**Figure 1:**
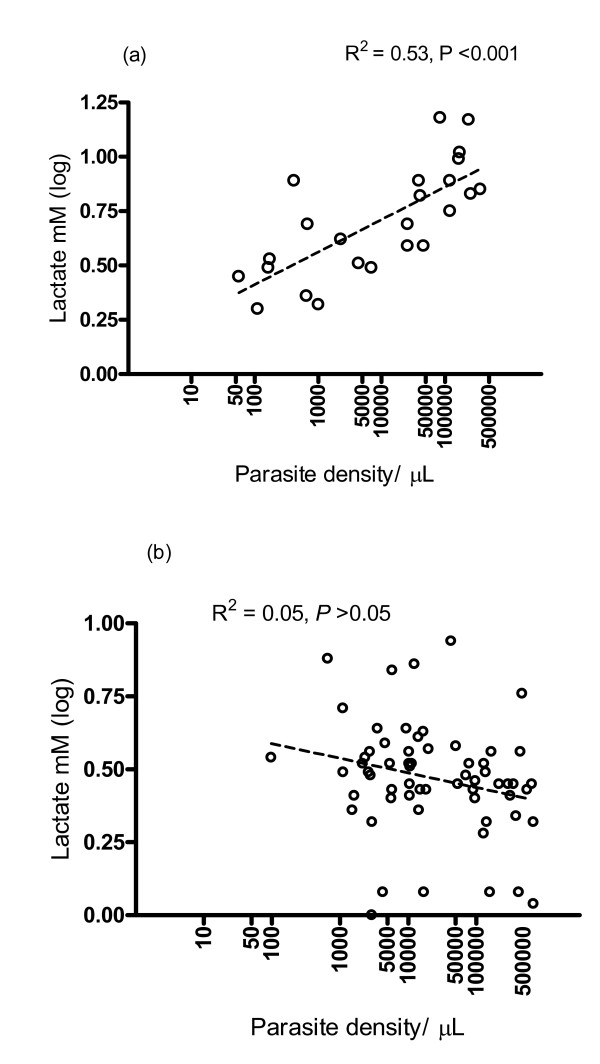
**Plasma lactate concentration and parasite density in children with acute malaria and severe anaemia (a) and non-severe anaemia (b)**. Parasite density is plotted on a log_e _scale. Dotted line represents regression line and R^2 ^is the coefficient of determination.

**Figure 2:**
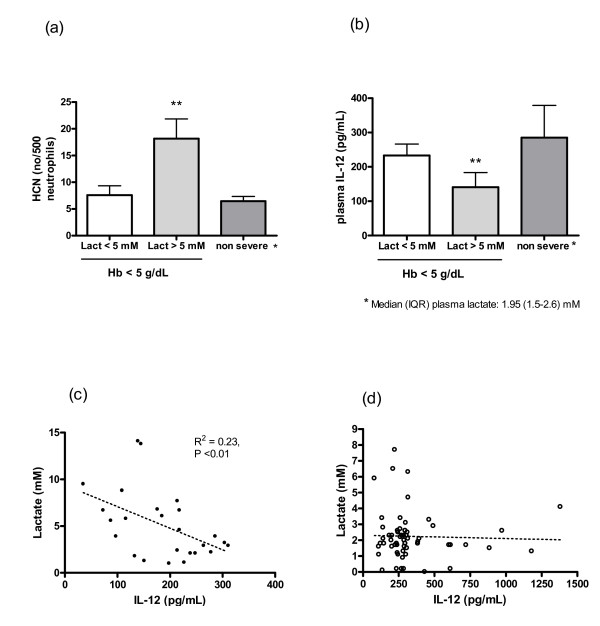
**Association of plasma lactate levels with HCN (a) and plasma IL-12 concentration (b) in children with severe malaria (N = 26) and non-severe malarial anaemia controls (N = 60). Scatter plot of the association of lactate concentration and IL-12 in children with severe anaemia and malaria (c) and non-severely anaemic controls with malaria (d)**. Bars show mean and error bars standard error of the mean. ** P < 0.01 (Mann-Whitney test). R^2 ^= coefficient of determination.

It was hypothesized that lactate levels in children with severe anaemia were associated with measures of parasite burden and with the prevailing cytokine levels. Indeed, lactate levels were associated with parasite density (*R*^2 ^= 0.53, *P *< 0.001) and with absolute numbers of HCNs (*r *= 0.60, *P *= 0.003) (Figure 1a and 2a). However, the same associations were not found in children with uncomplicated malarial anaemia (Figure 1b). Severe anaemia was associated with significantly higher plasma lactate levels and the median (IQR) plasma lactate was significantly higher (*P *= 0.01) in children with SMA and respiratory distress (8.8 [IQR 8.2–11.4] mM) than those without respiratory distress (3.5 [IQR 2.1–6.1] mM) (*P *= 0.01).

Only 46% of the children studied had plasma lactate concentrations higher than 5 mM. Children with hyperlactataemia (lactate > 5 mM) had significantly higher numbers of HCNs (*P *< 0.01) and lower plasma concentrations of IL-12 (*P *< 0.01) (Figure 2). The inverse association of plasma IL-12 and lactate was more pronounced in younger children (data not shown). Lactate was also associated with high levels of IL-10 (*r *= 0.54, *P *= 0.008) and negatively associated with IL-12 (*r *= -0.46, *P *= 0.02) but not with TNF-α or IFN-γ. No association was found between lactate levels and age or nutritional status (measured as weight-for-age Z-score).

The association of plasma lactate with HCNs and IL-12 was also measured in a group of children with uncomplicated malarial anaemia (N = 60). However, these associations were not significant in this group (*P *= 0.1 and *P *= 0.9, respectively).

Parasite density was closely associated with the numbers of Hz-containing neutrophils (*r *= 0.63, *P *< 0.001) and with levels of IL-10 (*r *= 0.75, *P *< 0.01). We therefore investigated whether the association of lactate concentration with HCNs, IL-12 and IL-10 was explained by parasite density. After controlling for parasite density, HCNs and IL-12 were still significantly associated with lactate concentration (*r *= 0.52, *P *= 0.016 and *r *= -0.55, *P *= 0.012, respectively). However, the association of lactate and IL-10 was no longer significant (*r *= 0.22, *P *= 0.34), suggesting that this association was mainly explained by the association of both lactate and IL-10 with parasite density.

All the variables linearly associated with lactate levels were included in a univariate regression analysis using lactate concentration as the dependent variable. Parasite density, HCNs and IL-12 were all strongly associated with lactate levels (*R*^2 ^= 0.28, *P *= 0.005; *R*^2 ^= 0.32, *P *= 0.003; *R*^2 ^= 0.23, *P *= 0.02, respectively). Due to the high degree of covariance and the sample size it was not possible to fit all the independent variables in a multiple regression model.

## Discussion

In this study, the association of the concentration of plasma lactate with parasite density and haemozoin in leucocytes and an inverse relationship with IL-12 concentration in children with severe malarial anaemia have been described. In previous studies, parasite density and Hz-containing neutrophils have been associated with disease severity but not with specific syndromes of severe malaria. The inverse relationship between IL-12 and lactate levels in children with malaria had not been described previously. These simple clinical associations may be important to guide further pathophysiological studies.

Following initial observations of an association between HCN and outcome [8] and both HCN and HCM with severe disease [11] two studies have shown an association between the number of HCM and anaemia and with plasma levels of TNF-α in a univariate analysis [12] and between the number of circulating HCM and a group of 26 children with Hb < 5 g/dL [10]. No studies had reported an association of HCN or HCM with increase in lactate concentration.

In this study, HCNs were associated with lactate concentration, but HCMs were not associated with lactate levels, even though HCNs and HCMs are closely correlated. It is possible that the different clearance times of HCMs (median, 9 days) and HCNs (median, 3 days) may partially explain these differences [7]. Thus, HCNs may reflect the recent levels of parasite burden and sequestration, contributing to micro-circulatory obstruction.

However, other explanations for the association of HCNs and lactate are plausible. The number of Hz containing-leucocytes may be a surrogate for the recent release of Hz content in the body and it is increasingly clear that Hz is not a "harmless" by-product of the digestion of Hb. Hz may cause widespread cellular dysfunction in endothelial cells, dendritic cells, monocytes/macrophages (reviewed in [20]) and uninfected erythrocytes [21] and so indirectly contribute to increased lactate levels.

Alternatively, the association of HCNs with lactate may reflect the induction of cytokine expression by Hz. This study and others [12] have found that HCNs are positively correlated with IL-10 and TNF-α and negatively with IL-12. This pattern of cytokine production is consistent with that observed *in vitro *in leucocytes fed with Hz [22].

The inverse association of lactate and IL-12 is intriguing. There is experimental evidence that IL-12 may enhance erythropoiesis [23] and correct anaemia in murine models of malaria [24]. This association is independent of parasite density and suggests that some effects other than the influence of IL-12 induction on the immune responses against the parasite are operating. One possibility is that IL-12 acts to increase the expression of inducible nitric oxide synthase (iNOS). NO might improve the oxygen delivery microcirculation in the face of low oxygen carrying capacity in the blood, poor blood flow due to increased red blood rigidity and obstruction of vessels by infected erythrocytes. While the role in NO in severe malaria remains unclear, there is clinical evidence that iNOS expression in increased in severe malaria and that NO is protective against severe malaria [25]. The results of this study would be consistent with the hypothesis that a protective IL-12/iNOS response could improve the delivery of oxygen to tissue by regulation of the microcirculation in severe malaria.

The roles of other cytokines in lactic acidosis are less clear in the present study. Although TNF-α has been previously associated with malarial severity [15], no significant association of TNF-α with lactate was found.

This study provides the first evidence of a positive association between Hz-containing leukocytes and lactate concentration. It is, therefore, possible that parasite density, Hz and IL-12 contribute to or counteract the development of lactic acidosis in severe malaria as elements of a common pathophysiological pathway. However, it was not possible to distinguish whether the association of HCNs and lactate represents an effect of Hz on cellular metabolism or function and/or an effect on cytokine secretion. More powerful clinical studies, using a multivariate analysis, and focused experimental and *ex vivo *studies should investigate how these factors contribute to the increase of lactate levels and identify potential therapeutic strategies against metabolic acidosis associated with severe malaria.

## competing interests

The author(s) declare that they have no competing interests.

## Authors' contributions

C. C-P contributed to the study design, protocols, preparation and examination of samples, collection of clinical data, analysis of data and preparation of the manuscript. O. K. contributed to the optimization of protocols and preparation and examination of clinical samples. B. L assisted in the preparation of laboratory protocols and supervised the laboratory work in Kenya. M.E, N. P, C. R. C. J. N, K. M and T. N. W contributed to the study design, supervision of recruitment and clinical work and the editing of the manuscript. D.J.R. contributed to the initiation of the project, experimental study design and protocols, data analysis and preparation of the manuscript.

## References

[B1] Krishna S, Waller DW, ter Kuile F, Kwiatkowski D, Crawley J, Craddock CF, Nosten F, Chapman D, Brewster D, Holloway PA (1994). Lactic acidosis and hypoglycaemia in children with severe malaria: pathophysiological and prognostic significance. Trans R Soc Trop Med Hyg.

[B2] English M, Sauerwein R, Waruiru C, Mosobo M, Obiero J, Lowe B, Marsh K (1997). Acidosis in severe childhood malaria. Qjm.

[B3] Marsh K, Forster D, Waruiru C, Mwangi I, Winstanley M, Marsh V, Newton C, Winstanley P, Warn P, Peshu N, Pasvol G, Snow RW (1995). Indicators of life-threatening malaria in African children. N Engl J Med.

[B4] Vander Jagt DL, Hunsaker LA, Campos NM, Baack BR (1990). D-lactate production in erythrocytes infected with *Plasmodium falciparum*. Mol Biochem Parasitol.

[B5] Dondorp AM, Angus BJ, Hardeman MR, Chotivanich KT, Silamut K, Ruangveerayuth R, Kager PA, White NJ, Vreeken J (1997). Prognostic significance of reduced red blood cell deformability in severe falciparum malaria. Am J Trop Med Hyg.

[B6] Field JWNJ, Hodgkin EP (1937). The prevention of malaria in the field by the use of quinine and atebrine. Quarterly Bulletin of the Health Organisation of the League of Nations.

[B7] Day NP, Pham TD, Phan TL, Dinh XS, Pham PL, Ly VC, Tran TH, Nguyen TH, Bethell DB, Nguyan HP, Tran TH, White NJ (1996). Clearance kinetics of parasites and pigment-containing leukocytes in severe malaria. Blood.

[B8] Nguyen PH, Day N, Pram TD, Ferguson DJ, White NJ (1995). Intraleucocytic malaria pigment and prognosis in severe malaria. Trans R Soc Trop Med Hyg.

[B9] Metzger WG, Mordmuller BG, Kremsner PG (1995). Malaria pigment in leucocytes. Trans R Soc Trop Med Hyg.

[B10] Lyke KE, Diallo DA, Dicko A, Kone A, Coulibaly D, Guindo A, Cissoko Y, Sangare L, Coulibaly S, Dakouo B, Taylor TE, Doumbo OK, Plowe CV (2003). Association of intraleukocytic *Plasmodium falciparum *malaria pigment with disease severity, clinical manifestations, and prognosis in severe malaria. Am J Trop Med Hyg.

[B11] Amodu OK, Adeyemo AA, Olumese PE, Gbadegesin RA (1998). Intraleucocytic malaria pigment and clinical severity of malaria in children. Trans R Soc Trop Med Hyg.

[B12] Luty AJ, Perkins DJ, Lell B, Schmidt-Ott R, Lehman LG, Luckner D, Greve B, Matousek P, Herbich K, Schmid D, Weinberg JB, Kremsner PG (2000). Low interleukin-12 activity in severe *Plasmodium falciparum *malaria. Infect Immun.

[B13] Casals-Pascual C, Kai O, Cheung JO, Williams S, Lowe B, Nyanoti M, Williams TN, Maitland K, Molyneux M, Newton CR, Peshu N, Watt SM, Roberts DJ (2006). Suppression of erythropoiesis in malarial anemia is associated with hemozoin in vitro and in vivo. Blood.

[B14] Malaguarnera L, Musumeci S (2002). The immune response to *Plasmodium falciparum *malaria. Lancet Infect Dis.

[B15] Kurtzhals JA, Adabayeri V, Goka BQ, Akanmori BD, Oliver-Commey JO, Nkrumah FK, Behr C, Hviid L (1998). Low plasma concentrations of interleukin 10 in severe malarial anaemia compared with cerebral and uncomplicated malaria. Lancet.

[B16] Malaguarnera L, Imbesi RM, Pignatelli S, Simpore J, Malaguarnera M, Musumeci S (2002). Increased levels of interleukin-12 in *Plasmodium falciparum *malaria: correlation with the severity of disease. Parasite Immunol.

[B17] Pichyangkul S, Saengkrai P, Webster HK (1994). *Plasmodium falciparum *pigment induces monocytes to release high levels of tumor necrosis factor-alpha and interleukin-1 beta. Am J Trop Med Hyg.

[B18] Keller CC, Kremsner PG, Hittner JB, Misukonis MA, Weinberg JB, Perkins DJ (2004). Elevated nitric oxide production in children with malarial anemia: hemozoin-induced nitric oxide synthase type 2 transcripts and nitric oxide in blood mononuclear cells. Infect Immun.

[B19] Molyneux ME, Taylor TE, Wirima JJ, Borgstein A (1989). Clinical features and prognostic indicators in paediatric cerebral malaria: a study of 131 comatose Malawian children. Q J Med.

[B20] Urban BC, Roberts DJ (2002). Malaria, monocytes, macrophages and myeloid dendritic cells: sticking of infected erythrocytes switches off host cells. Curr Opin Immunol.

[B21] Omodeo-Sale F, Motti A, Dondorp A, White NJ, Taramelli D (2005). Destabilisation and subsequent lysis of human erythrocytes induced by *Plasmodium falciparum *haem products. Eur J Haematol.

[B22] Mordmuller B, Turrini F, Long H, Kremsner PG, Arese P (1998). Neutrophils and monocytes from subjects with the Mediterranean G6PD variant: effect of *Plasmodium falciparum *hemozoin on G6PD activity, oxidative burst and cytokine production. Eur Cytokine Netw.

[B23] Dybedal I, Larsen S, Jacobsen SE (1995). IL-12 directly enhances in vitro murine erythropoiesis in combination with IL-4 and stem cell factor. J Immunol.

[B24] Mohan K, Stevenson MM (1998). Interleukin-12 corrects severe anemia during blood-stage *Plasmodium chabaudi *AS in susceptible A/J mice. Exp Hematol.

[B25] Anstey NM, Weinberg JB, Hassanali MY, Mwaikambo ED, Manyenga D, Misukonis MA, Arnelle DR, Hollis D, McDonald MI, Granger DL (1996). Nitric oxide in Tanzanian children with malaria: inverse relationship between malaria severity and nitric oxide production/nitric oxide synthase type 2 expression. J Exp Med.

